# Direct comparison of low-dose-rate brachytherapy versus radical prostatectomy using the surgical definition of biochemical recurrence for patients with intermediate-risk prostate cancer

**DOI:** 10.1186/s13014-022-02046-x

**Published:** 2022-04-11

**Authors:** Hideyasu Tsumura, Nobumichi Tanaka, Tomohiko Oguchi, Takuya Owari, Yasushi Nakai, Isao Asakawa, Kazuyoshi Iijima, Haruaki Kato, Iwao Hashida, Ken-ichi Tabata, Takefumi Satoh, Hiromichi Ishiyama

**Affiliations:** 1 Department of Urology, Kitasato University School of Medicine, 1-15-1 Kitasato Minami-ku, Sagamihara, Kanagawa 252-0374 Japan; 2 Department of Urology, Nara Medical University, Kashihara, Japan; 3 Department of Urology, Nagano Municipal Hospital, Nagano, Japan; 4 Department of Radiation Oncology, Nara Medical University, Kashihara, Japan; 5 Department of Radiation Therapy, Nagano Municipal Hospital, Nagano, Japan; 6 Department of Radiation Oncology, Kitasato University School of Medicine, Sagamihara, Japan

**Keywords:** Brachytherapy, Intermediate risk, Prostate cancer, Radical prostatectomy

## Abstract

**Background:**

We compared the oncological outcomes of patients who received seed brachytherapy (SEED-BT) with those who received radical prostatectomy (RP) for intermediate-risk prostate cancer.

**Methods:**

Candidates were patients treated with either SEED-BT (n = 933) or RP (n = 334). One-to-one propensity score matching was performed to adjust the patients’ backgrounds. We compared the biochemical recurrence (BCR)-free rate using the Phoenix definition (prostate-specific antigen [PSA] nadir plus 2 ng/mL) for SEED-BT and the surgical definition (PSA cut-off value of 0.2 ng/mL) for RP. We also directly compared the BCR-free rates using the same PSA cut-off value of 0.2 ng/mL for both SEED-BT and RP.

**Results:**

In the propensity score-matched analysis with 214 pairs, the median follow-up treatment was 96 months (range 1–158 months). Fifty-three patients (24.7%) were treated with combined SEED-BT and external-beam radiotherapy. Forty-three patients (20.0%) received salvage radiotherapy after RP. Comparing the BCR-free rate using the above definitions for SEED-BT and RP showed that SEED-BT yielded a significantly better 8-year BCR-free rate than did RP (87.4% vs. 74.3%, hazard ratio [HR] 0.420, 95% confidence interval [CI] 0.273–0.647). Comparing the 8-year BCR-free rate using the surgical definition for both treatments showed no significant difference between the two treatments (76.7% vs. 74.3%, HR 0.913, 95% CI 0.621–1.341). SEED-BT had a significantly better 8-year salvage hormonal therapy-free rate than did RP (92.0% vs. 85.6%, HR 0.528, 95% CI 0.296–0.942, *P* = 0.030). The 8-year metastasis-free survival rates (98.5% vs. 99.0%, HR 1.382, 95% CI 0.313–6.083, *P* = 0.668) and overall survival rates (91.9% vs. 94.6%, HR 1.353, 95% CI 0.690–2.650) did not significantly differ between the treatments.

**Conclusions:**

The BCR-free rates did not significantly differ between patients treated with SEED-BT and those treated with RP for intermediate-risk prostate cancer even when they were directly compared using the surgical definition for BCR. SEED-BT and RP can be adequately compared for oncological outcomes.

**Supplementary Information:**

The online version contains supplementary material available at 10.1186/s13014-022-02046-x.

## Background

Intermediate-risk prostate cancer affects patients with heterogeneous oncological outcomes and has various treatment options [1]. For patients who desire curative treatment, permanent seed brachytherapy (SEED-BT) and radical prostatectomy (RP) are widely accepted definitive therapeutic options [2–5]. Clinicians should provide comparative information about these treatment modalities and their therapeutic effects to help their patients make informed decisions. Although conducting prospective randomized controlled studies comparing SEED-BT and RP is not feasible [6, 7], several retrospective studies have compared the oncological outcomes between these treatments [8, 9]. These studies experienced some difficulties in making these comparisons because they proposed different definitions of biochemical recurrence (BCR) for each treatment [10, 11]. Different patient characteristics at baseline and the impacts of surgical techniques on margin control during RP also made the comparison difficult [12].

In this study, we compared the oncological outcomes of intermediate-risk prostate cancer treatments between SEED-BT and RP using propensity score matching, which minimizes imbalances in patient characteristics between treatment modalities. Because the definitions of BCR differ between the two treatment modalities, we compared the BCR using the same definition, with the surgical definition of the prostate-specific antigen (PSA) cut-off value being 0.2 ng/mL for both SEED-BT and RP. We also compared the effectiveness of both treatment modalities by excluding patients with positive surgical margins for RP and their matched pairs for SEED-BT to eliminate the effect of surgical techniques on oncological outcomes.

## Materials and methods

### Patient selection

Candidates for the present study were patients with intermediate-risk prostate cancer who underwent SEED-BT plus or minus the combination of external-beam radiotherapy (EBRT) and RP at three tertiary hospitals between January 2006 and December 2011. RP was performed via either the open retropubic approach or laparoscopic surgery. Patients with no evidence of BCR and < 4 years of follow-up were excluded. Patients who died from any cause or who developed BCR within 4 years post-treatment were included. Patients who received neoadjuvant hormonal therapy (NHT) for > 12 months or any adjuvant hormonal therapy were excluded. No fixed protocol was used for NHT for patients undergoing SEED-BT and RP. The main reason for using NHT was to reduce patients’ gland size (prostate gland volumes > 50 cm^3^), which can make SEED-BT technically more difficult.

### Treatment protocol for SEED-BT at each institution

We previously described the treatment protocol for SEED-BT with or without the combination of EBRT [13, 14]. Briefly, for the SEED-BT treatments, most patients at the three institutions were treated using an intraoperative planning method by modified peripheral loading techniques using loose seeds. Iodine-125 was used for all patients. The doses were defined using TG43 criteria. Computed tomography-based dosimetric analysis was performed to calculate the D90, V100, and V150 dosage results 1 month after SEED-BT. Patients were treated with SEED-BT alone at a prescribed dose of 145 Gy or 160 Gy according to each institution’s treatment protocol.

For patients treated with a combination of SEED-BT and EBRT at institution A, the prescribed dose of SEED-BT was 110 Gy. The target portion of EBRT was determined 1 month after seed implant, and the patients received 45 Gy (in 25 fractions of 1.8 Gy/fraction) using 10 MV of photon energy (three-dimensional conformal radiotherapy). The clinical target volume (CTV) for EBRT was defined as the prostate. A planning target volume (PTV) for EBRT was created by adding an 8-mm margin all around the CTV except posteriorly, where it was limited to 3 mm. At institution B, the prescribed dose of SEED-BT for the combined therapy was 100 Gy. EBRT was finished 2 weeks before seed implant, and the patients received 46 Gy (in 23 fractions of 2 Gy/fraction) using 10 MV of photon energy (three-dimensional conformal radiotherapy). The CTV for EBRT was defined as the prostate and one-third of the proximal seminal vesicle. A PTV for EBRT was created by adding a 10-mm margin all around the CTV except posteriorly, where it was limited to 6 mm. At institution C, no patients were treated with a combination of SEED-BT and EBRT.

### Definitions of outcome measurements

We compared the BCR-free rate between the two treatment modalities using the Phoenix definition for SEED-BT and the surgical definition for RP. We also compared the BCR-free rate between the two treatment modalities using the surgical definition for both SEED-BT and RP. For the Phoenix definition (PSA nadir plus 2 ng/mL), patients with an increase of ≥ 2 ng/mL above the nadir PSA were considered to have BCR. However, a spontaneous decrease of < 2 ng/mL plus nadir PSA occurred in some patients who were defined as having BCR, but these patients were not considered to have BCR. Regarding the surgical definition (PSA cut-off: 0.2 ng/mL), patients were defined as having BCR if the PSA value increased above 0.2 ng/mL on two consecutive measurements after the PSA fell to < 0.2 ng/mL. However, spontaneous decreases of < 0.2 ng/mL for RP and SEED-BT occurred in some patients defined as having BCR, but these patients were not considered to have BCR [14]. If the PSA value never fell below 0.2 ng/mL, the patient was defined as having BCR at the date of RP or the initiation of radiotherapy. Both distant metastasis and regional lymph node metastasis were classified as metastasis in the analysis for metastasis-free survival.

Biological effective dose equations were used with an α/β ratio of 2 as per Stock et al. [15]. The National Comprehensive Cancer Network 2019 guidelines (version 4) were used to identify the intermediate-risk patients and categorize them as favorable or unfavorable.

### Statistical analyses

Characteristics were compared between patients who underwent SEED-BT and those who underwent RP using the chi-square test for categorical variables and the Wilcoxon rank-sum test for continuous variables. The propensity score-matched analysis was performed using the teffects psmatch function (Stata Press, 2013) [16, 17]. We estimated the average treatment effects of SEED-BT on BCR using the same function. Age, PSA at diagnosis, Gleason grade group, clinical stage, and positive biopsy core rates were the variables used to calculate the propensity scores. A propensity score analysis with 1:1 matching was performed with nearest-neighbor matching. Kaplan–Meier methods were used to estimate the 8-year survival rates, and the differences were assessed with the log-rank statistic.

Mantel–Haenszel hazard ratios (HRs) were calculated for the outcomes. Univariate and multivariate Cox proportional hazards regression models were performed to evaluate the risk factors for BCR and salvage hormonal therapy for each treatment modality.

Differences were considered statistically significant at *P* < 0.05. All reported *P*-values are two-sided. All analyses were performed with Stata, version 15 (Stata Corp., College Station, TX, USA) and GraphPad Prism, version 8 (GraphPad Software, Inc., La Jolla, CA, USA).

## Results

**Fig. 1:**
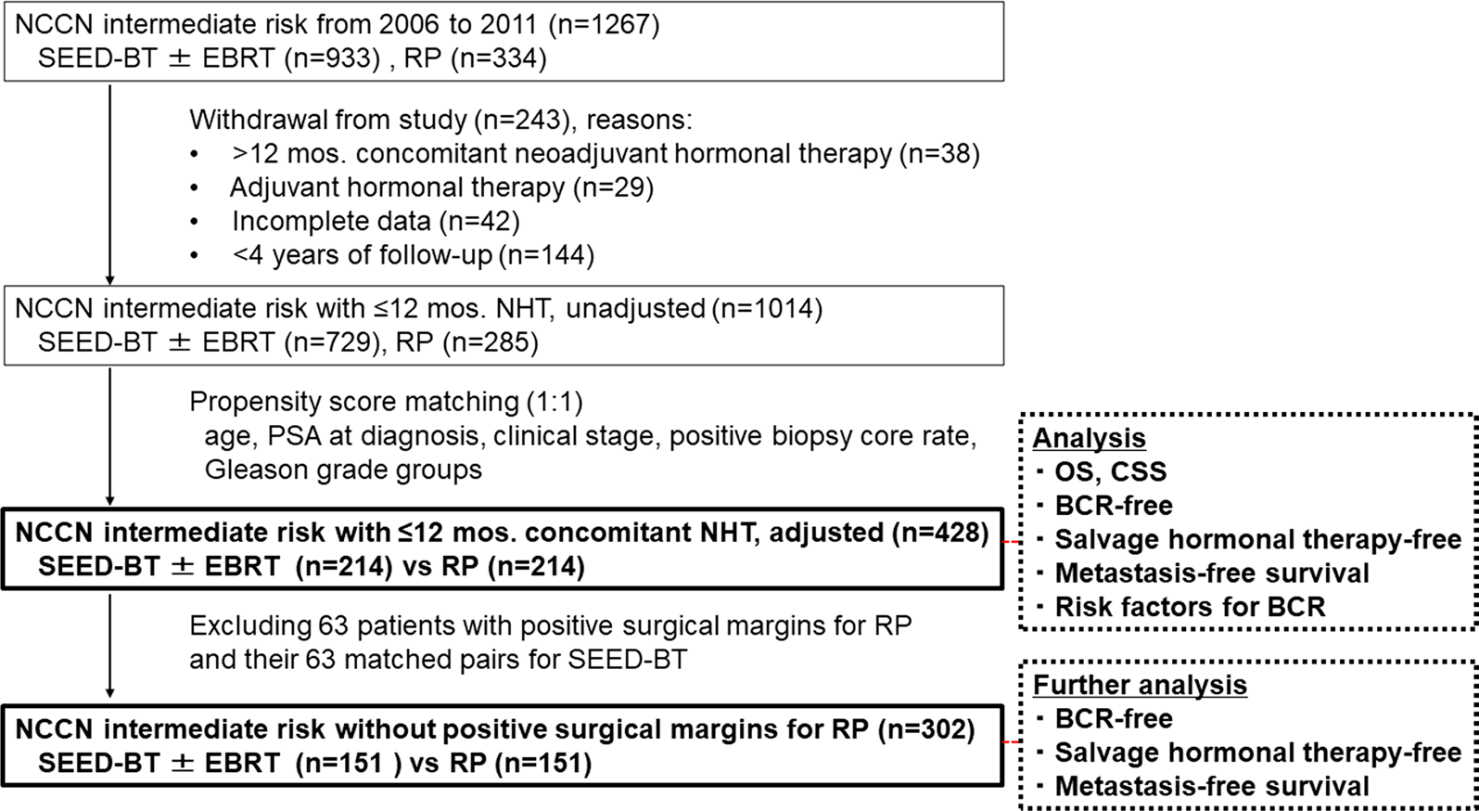
Patient selection. Abbreviations; BCR, biochemical recurrence; CSS, cancer-specific survival; EBRT, external-beam radiotherapy; NCCN, National Comprehensive Cancer Network guidelines (2019, version 4); NHT, neoadjuvant hormonal therapy; OS, overall survival; PSA, prostate-specific antigen; SEED-BT, seed brachytherapy; RP, radical prostatectomy

Figure 1 shows the patient selection process. In total, 1267 patients with intermediate-risk prostate cancer were treated with either SEED-BT (n = 933) or RP (n = 334) at three institutions. Among them, 243 patients were excluded from the study; Fig. 1 provides the reasons for their exclusion. Finally, 1014 patients met the inclusion criteria. One-to-one propensity score matching was performed using the clinical data for the 1014 patients, which yielded 214 pairs. For further analysis, 63 patients with positive surgical margins for RP and 63 matched pairs who underwent SEED-BT were excluded to eliminate the effect of surgical technique on oncological outcomes.

Table 1 shows the patients’ characteristics after propensity score matching. The median follow-up was 96 months (range 1–158 months). Forty-three patients (20.0%) received salvage radiotherapy for the prostate bed after RP. Fifty-three patients (24.7%) received a combination of EBRT for SEED-BT. Sixty-three patients who underwent RP (29.4%) had positive surgical margins. Ninety-three patients who received RP (43.4%) underwent laparoscopic RP.

**Table 1:** Patient characteristics, adjusted

Variables	SEED-BT (n = 214)	RP (n = 214)	*P*
Age, median (range), years	69 (48–82)	68 (52–79)	0.204
PSA at diagnosis, median (range), ng/mL	7.9 (2.6–18.5)	7.3 (2.9–19.8)	0.697
Clinical stage, number (%)			0.562
T1c	101 (47.1%)	107 (50.0%)	
T2a–c	113 (52.9%)	107 (50.0%)	
Gleason grade, number (%)			0.770
1	41 (19.2%)	36 (16.8%)	
2	112 (52.3%)	112 (52.3%)	
3	61 (28.5%)	66 (30.9%)	
Positive biopsy core rate, number (%)			0.552
< 34%	128 (59.8%)	134 (62.6%)	
≥ 34%	86 (40.2%)	80 (37.4%)	
Favorable intermediate-risk, number (%)	101 (47.2%)	100 (46.7%)	0.923
Follow-up, median (range), mos	96 (1–153)	94 (11–158)	0.499
Neoadjuvant hormonal therapy yes, number (%)	65 (30.3%)	9 (4.2%)	–
Adjuvant hormonal therapy yes, number (%)	0 (0.0%)	0 (0.0%)	–
Salvage radiotherapy, number (%)	–	43 (20.0%)	–
Positive surgical margin, number (%)	–	63 (29.4%)	–
Open surgery (n = 121), number (%)		42 (34.7%)	
Laparoscopic surgery (n = 93), number (%)		21 (22.5%)	
Combined EBRT, number (%)	53 (24.7%)	–	–
Favorable intermediate-risk	12 (5.60%)		
Unfavorable intermediate-risk	41 (19.1%)		
Prostate D90 at 1 month, Gy			
SEED-BT alone	185.6 (141.0–255.0)	–	
Combined EBRT	124.4 (99.4–180.8)	–	
Prostate V100 at 1 month, Gy			
SEED-BT alone	97.9% (80.1–100)	–	
Combined EBRT	96.9% (90.0–99.3)	–	
Prostate V150 at 1 month, Gy			
SEED-BT alone	69.2% (23.4–97.3)	–	
Combined EBRT	62.4% (42.6–84.8)	–	
BED, Gy2	218.0 (147.9–284.1)	–	

Prostate D90 indicates minimal dose received by 90% of prostate gland at 1 month. Prostate V100 and V150 indicates percentage of prostate gland volume that received 100% and 150% of the prescribed dose, respectively, at 1 month

BED, biochemical effective dose; EBRT, external-beam radiotherapy; PSA, prostate-specific antigen; SEED-BT, seed brachytherapy; RP, radical prostatectomy

### Outcomes

**Fig. 2:**
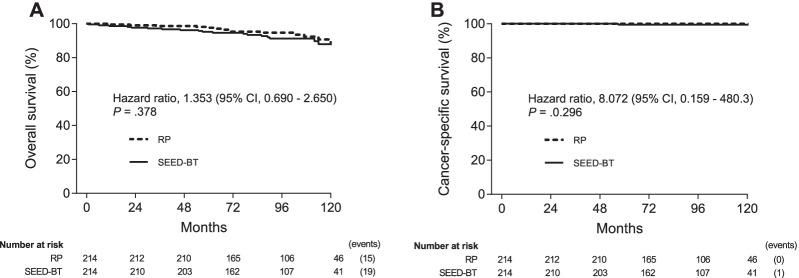
Kaplan–Meier estimates of overall survival (**A**) and prostate cancer-specific survival (**B**) for SEED-BT and RP. Abbreviations; CI, confidence interval; RP, radical prostatectomy; SEED-BT, seed brachytherapy

Nineteen patients who received SEED-BT and 15 who underwent RP died during follow-up. One who received SEED-BT died of prostate cancer (Additional file 1: Table S1). Neither the 8-year overall survival rates (91.9% vs 94.6%, HR: 1.353, 95% confidence interval [CI] 0.690–2.650, *P* = 0.378; Fig. 2A) nor the prostate cancer-specific survival rates (99.4% vs 100%, HR 8.072, 95% CI 0.159–408.3, *P* = 0.296; Fig. 2B) differed significantly between patients who received SEED-BT vs those who received RP.

**Fig. 3:**
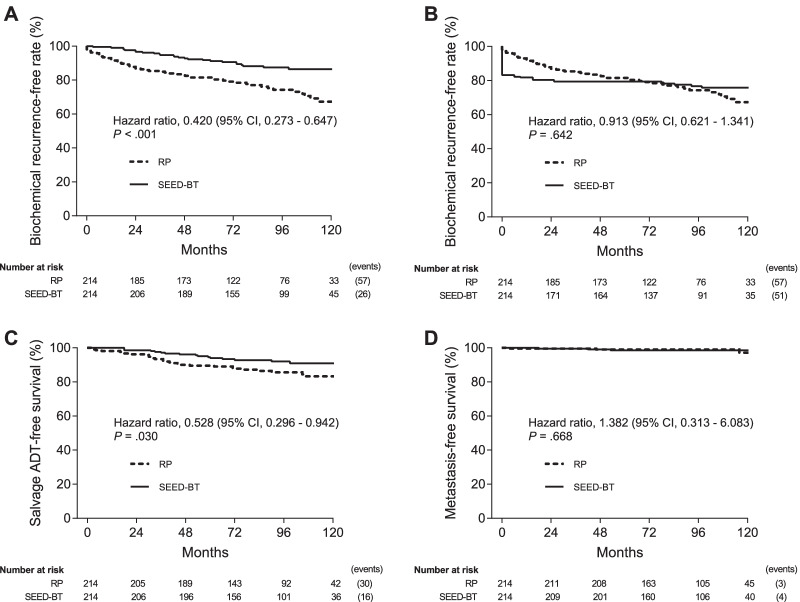
Kaplan–Meier estimates of the biochemical recurrence-free rate using the Phoenix definition (PSA nadir plus 2 ng/mL) for SEED-BT and the surgical definition (PSA cut-off value of 0.2 ng/mL) for RP (**Ab**. Kaplan–Meier estimates of the biochemical recurrence-free rate using the surgical definition (PSA cut-off value of 0.2 ng/mL) for both SEED-BT and RP (**B**). Salvage hormonal therapy-free survival rate (**C**) and metastasis-free survival rate (**D**) for SEED-BT and RP. Abbreviations: CI, confidence interval; RP, radical prostatectomy; SEED-BT, seed brachytherapy

When the BCR-free rates were calculated using the Phoenix definition for SEED-BT and the surgical definition for RP, SEED-BT had a significantly better 8-year BCR-free rate than did RP (87.4% vs 74.3%, HR 0.420, 95% CI 0.273–0.647, *P* < 0.001; Fig. 3A). When the BCR-free rates were calculated with the surgical definition of a PSA cut-off value of 0.2 ng/mL for both SEED-BT and RP, the 8-year BCR-free rates did not significantly differ between the two treatments (76.4% vs 74.3%, HR 0.913, 95% CI 0.621–1.341, *P* = 0.642; Fig. 3B). SEED-BT had a significantly better 8-year salvage hormonal therapy-free rate than did RP (92.0% vs 85.6%, HR 0.528, 95% CI 0.296–0.942, *P* = 0.030; Fig. 3C). The 8-year metastasis-free survival rates did not significantly differ between the two treatments (98.5% vs 99.0%, HR 1.382, 95% CI 0.313–6.083, *P* = 0.668; Fig. 3D).

**Fig. 4:**
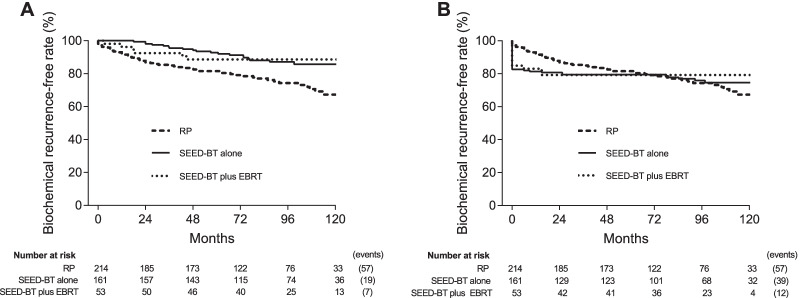
Subgroup analysis for the biochemical recurrence-free rate using the Phoenix definition (PSA nadir plus 2 ng/mL) for SEED-BT and the surgical definition (PSA cut-off value of 0.2 ng/mL) for RP (**A**). Subgroup analysis for the biochemical recurrence-free rate using the surgical definition (PSA cut-off value of 0.2 ng/mL) for both SEED-BT and RP (**B**). Abbreviations: RP, radical prostatectomy; SEED-BT, seed brachytherapy; EBRT, external-beam radiotherapy

Figure 4A showed the subgroup analysis for the BCR-free rates calculated with the Phoenix definition for SEED-BT and the surgical definition for RP. SEED-BT alone had a significantly better 8-year BCR-free rate than did RP (87.1% vs 74.3%, HR 0.425, 95% CI 0.271–0.669, *P* < 0.001). SEED-BT plus EBRT was likely to have a better 8-year BCR-free rate than did RP but the BCR-free rates did not statistically differ between the two treatments (88.6% vs 74.3%, HR 0.551, 95% CI 0.295–1.013, *P* = 0.055). The 8-year BCR-free rates did not significantly differ between SEED-BT alone vs SEED-BT plus EBRT (87.1% vs 88.6%, HR 0.848, 95% CI 0.344–2.093, *P* = 0.721). Figure 4B showed the subgroup analysis for the BCR-free rates calculated with the surgical definition for both SEED-BT and RP. The 8-year BCR-free rates did not significantly differ between SEED-BT alone vs RP (75.8% vs 74.3%, HR 0.916, 95% CI 0.607–1.383, *P* = 0.678), SEED-BT plus EBRT vs RP (79.2% vs 74.3%, HR 0.941, 95% CI 0.508–1.741, *P* = 0.846), and SEED-BT alone vs SEED-BT plus EBRT (75.8% vs 79.2%, HR 1.054, 95% CI 0.532–2.085, *P* = 0.880), respectively.

**Fig. 5:**
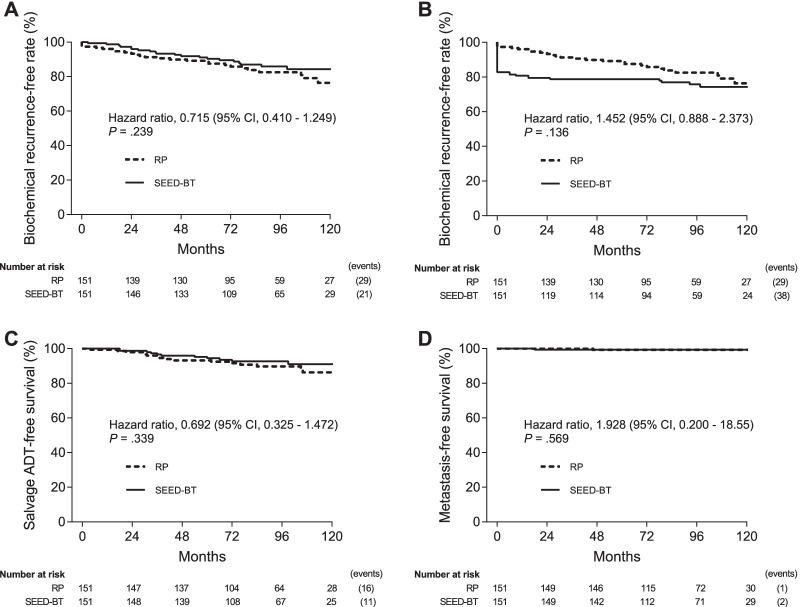
To eliminate the effects of surgical artifacts on clinical outcomes, 63 patients with positive surgical margins for RP and their 63 matched pairs for SEED-BT were excluded from the analyses. Kaplan–Meier estimates of the biochemical recurrence-free rate were calculated using the Phoenix definition (PSA nadir plus 2 ng/mL) for SEED-BT and the surgical definition (PSA cut-off value of 0.2 ng/mL) for RP (**A**). Kaplan–Meier estimates of the biochemical recurrence-free rate using the surgical definition (PSA cut-off value of 0.2 ng/mL) for both SEED-BT and RP (**B**). Salvage hormonal therapy-free survival rate (**C**) and metastasis-free survival rate (**D**) for SEED-BT and RP. Abbreviations: CI, confidence interval; RP, radical prostatectomy; SEED-BT, seed brachytherapy

To eliminate the effect of surgical technique on clinical outcomes, we performed further analyses that excluded 63 patients with positive surgical margins for RP and their 63 matched pairs for SEED-BT (Table 2). Here, the 8-year BCR-free rate did not significantly differ between the two treatments using the Phoenix definition for SEED-BT and the surgical definition for RP (85.9% vs 82.5%, HR: 0.715, 95% CI 0.410–1.249, *P* = 0.239; Fig. 5A). Further, the 8-year BCR-free rate did not significantly differ between the two treatments using the surgical definition of a PSA cut-off value of 0.2 ng/mL for both SEED-BT and RP (75.7% vs 82.5%, HR: 1.452, 95% CI: 0.888–2.373, *P* = 0.136; Fig. 5B). Additionally, no significant differences were found in the 8-year salvage hormonal therapy-free rates (92.7% vs 89.6%, HR 0.692, 95% CI 0.325–1.472, *P* = 0.339; Fig. 5C) or metastasis-free survival rates (99.3% vs 99.3%, HR 1.928, 95% CI 0.200–18.55, *P* = 0.569; Fig. 5D) between the two treatments.

**Table 2:** Patient characteristics excluding 63 patients with positive surgical margins for RP and their 63 matched pairs for SEED-BT

Variables	SEED-BT (n = 151)	RP (n = 151)	*P*
Age, median (range), years	69 (48–82)	68 (53–79)	0.214
PSA at diagnosis, median (range), ng/mL	8.3 (2.6–18.0)	6.3 (2.9–19.8)	0.253
Clinical stage, number (%)			1.000
T1c	84 (55.6%)	84 (55.6%)	
T2a–c	67 (44.4%)	67 (44.4%)	
Gleason grade, number (%)			0.472
1	30 (19.9%)	23 (15.2%)	
2	82 (54.3%)	82 (54.3%)	
3	39 (25.8%)	46 (30.5%)	
Positive biopsy core rate, number (%)			0.552
< 34%	92 (60.9%)	97 (64.2%)	
≥ 34%	59 (39.1%)	54 (35.8%)	
Favorable intermediate-risk, number (%)	74 (49.0%)	77 (51.0%)	0.730
Follow-up, median (range), mos	93 (1–153)	92 (11–153)	0.434
Neoadjuvant hormonal therapy yes, number (%)	46 (30.5%)	8 (5.2%)	–
Adjuvant hormonal therapy yes, number (%)	0 (0.0%)	0 (0.0%)	–
Salvage radiotherapy, number (%)	–	23 (15.2%)	–
Positive surgical margin, number (%)	–	0 (0.0%)	–
Open surgery (n = 79), number (%)		0 (0.0%)	
Laparoscopic surgery (n = 72), number (%)		0 (0.0%)	
Combined EBRT, number (%)	39 (25.8%)	–	–
Favorable intermediate-risk	8 (7.9%)		
Unfavorable intermediate-risk	31 (27.1%)		
Prostate D90 at 1 month, Gy			
SEED-BT alone	185.3 (141.0–225.4)	–	
Combined EBRT	121.2 (99.4–180.8)	–	
Prostate V100 at 1 month, Gy			
SEED-BT alone	98.0% (80.1–100)	–	
Combined EBRT	96.4% (90.0–99.3)	–	
Prostate V150 at 1 month, Gy			
SEED-BT alone	68.2% (23.4–97.3)	–	
Combined EBRT	62.2% (43.6–84.9)	–	
BED, Gy2	204.8 (147.9.–284.1)	–	

Prostate D90 indicates minimal dose received by 90% of prostate gland at 1 month. Prostate V100 and V150 indicates percentage of prostate gland volume that received 100% and 150% of the prescribed dose, respectively, at 1 month

BED, biochemical effective dose; EBRT, external-beam radiotherapy; PSA, prostate-specific antigen; SEED-BT, seed brachytherapy; RP, radical prostatectomy

Table 3 shows the risk factors for BCR using the Phoenix definition and salvage hormonal therapy for SEED-BT. We detected no pre- or post-treatment risk factors for BCR or salvage hormonal therapy for SEED-BT. Table 4 shows the risk factors for BCR using the surgical definition and salvage hormonal therapy for RP. A higher PSA at diagnosis (HR 1.081, 95% CI 1.015–1.152), positive surgical margins (HR 2.169, 95% CI 1.218–3.865) and pathological T3-4 (HR 1.847, 95% CI 1.059–3.222) independently predicted BCR for patients who underwent RP. Gleason grade groups at biopsy (HR 2.318, 95% CI 1.199–4.479), pathological T3–4 (HR 2.531, 95% CI 1.185–5.402) and pathological Gleason grade groups (HR 1.448, 95% CI 1.025–2.045) independently predicted the use of salvage hormonal therapy for patients who underwent RP.

**Table 3:** Risk factors for biochemical recurrence using the Phoenix definition and salvage hormonal therapy in patients treated with SEED-BT

Variables	Univariate		
	HR	95% CI	*P*
*Risk factors for biochemical recurrence*			
Age, years	1.011	0.957–1.068	0.688
PSA at diagnosis, ng/mL	1.088	0.985–1.201	0.094
Clinical stage, T2a–c versus 1c	0.704	0.325–1.526	0.375
Gleason grade groups at biopsy	0.823	0.472–1.436	0.494
Positive biopsy core rate, ≥ 34% versus < 34%	0.774	0.344–1.740	0.536
Unfavorable versus favorable	1.413	0.641–3.117	0.391
BED < 200 Gy2 versus ≥ 200 Gy2	0.636	0.294–1.378	0.252
EBRT yes versus no	1.170	0.491–2.791	0.722
*Risk factors for salvage hormonal therapy*			
Age, years	1.032	0.959–1.110	0.394
PSA at diagnosis, ng/mL	0.928	0.799–1.079	0.334
Clinical stage, T2a–c versus 1c	1.437	0.521–3.962	0.482
Gleason grade groups at biopsy	0.931	0.457–1.896	0.846
Positive biopsy core rate, ≥ 34% versus < 34%	1.147	0.427–3.081	0.785
Unfavorable versus favorable	1.392	0.505–3.833	0.522
BED < 200 Gy2 versus ≥ 200 Gy2	0.964	0.358–2.590	0.942
EBRT yes versus no	1.427	0.495–4.108	0.510

The cut-off value for defining biochemical recurrence was nadir + 2 ng/mL (Phoenix definition)

Unfavorable and favorable indicate unfavorable intermediate risk and favorable intermediate risk, respectively (National Comprehensive Cancer Network 2019 guidelines, version 4)

BED, biochemical effective dose; EBRT, external-beam radiotherapy; PSA, prostate-specific antigen; SEED-BT, seed brachytherapy; RP, radical prostatectomy

**Table 4:** Risk factors for biochemical recurrence using the surgical definition and salvage hormonal therapy in patients treated with radical prostatectomy

Variables	Univariate			Multivariate		
	HR	95%CI	*P*	HR	95%CI	*P*
*Risk factors for biochemical recurrence*						
Age, years	0.967	0.925–1.012	0.163			
PSA at diagnosis, ng/mL	1.112	1.049–1.179	< 0.001	1.081	1.015–1.152	0.015
Clinical stage, T2a-c versus 1c	1.791	1.052–3.051	0.032			
Gleason grade groups at biopsy	1.528	1.015–2.302	0.042			
Positive biopsy core rate, ≥ 34% versus < 34%	1.012	0.550–1.736	0.964			
Unfavorable versus favorable	1.808	1.052–3.109	0.032			
Surgical margin, positive versus negative	3.191	1.874–5.433	< 0.001	2.169	1.218–3.865	0.009
Pathological T3–4 versus T0–2	2.491	1.470–4.223	0.001	1.847	1.059–3.222	0.030
Pathological Gleason grade groups	1.517	1.200–1.918	0.001	1.240	0.965–1.593	0.092
*Risk factors for salvage hormonal therapy*						
Age, years	0.986	0.924–1.052	0.687			
PSA at diagnosis, ng/mL	1.126	1.040–1.220	0.003			
Clinical stage, T2a–c versus 1c	3.010	1.339–6.765	0.008			
Gleason grade groups at biopsy	3.123	1.661–5.872	< 0.001	2.318	1.199–4.479	0.012
Positive biopsy core rate, ≥ 34% versus < 34%	1.126	0.542–2.340	0.749			
Unfavorable versus favorable	3.729	1.523–9.125	0.004			
Surgical margin, positive versus negative	2.213	1.080–4.536	0.030	1.610	0.762–3.401	0.211
Pathological T3–4 versus T0–2	4.051	1.165–8.352	< 0.001	2.531	1.185–5.402	0.016
Pathological Gleason grade groups	1.917	1.425–2.579	< 0.001	1.448	1.025–2.045	0.035

The cut-off value for defining biochemical recurrence was a PSA of 0.2 ng/mL

PSA, prostate-specific antigen

## Discussion

We used propensity score matching to evaluate and compare the oncological outcomes of patients with intermediate-risk prostate cancer who underwent SEED-BT with those who underwent RP. The BCR-free rates did not significantly differ between the two treatment modalities even when the BCR-free rates were evaluated using the same definition of a PSA cut-off value of 0.2 ng/mL for both treatments. When the analysis was performed with the assumption that no RP patients had positive surgical margins, the oncological outcomes still did not differ between the two treatment modalities. Additionally, the overall survival rate and prostate cancer-specific mortality rate did not significantly differ between the two treatments. Hence, SEED-BT and RP can be adequately compared for the oncological outcomes of intermediate-risk prostate cancer.

Grim et al. [2] performed a comparative analysis of PSA failure-free survival outcomes for patients with non-metastatic prostate cancer after radical therapy. For intermediate-risk patients, these authors reported a higher average BCR-free survival rate for SEED-BT than for RP. Goy et al. [9] compared the 10-year BCR-free rates between SEED-BT ± EBRT and RP for patients with intermediate-risk prostate cancer. When the BCR-free rates were calculated using the Phoenix definition for SEED-BT and the surgical definition for RP, SEED-BT yielded significantly higher 10-year BCR-free rates than did RP (80.2% vs 57.1%, *P* = 0.0003). Additionally, compared with SEED-BT, RP had a higher incidence of patients who received some form of salvage therapy. Our results were consistent with these reports. Compared with SEED-BT, RP may have a higher incidence of patients considered to have BCR and consequently need salvage therapies more frequently when the individual definition of PSA failure is used in daily clinical practice.

The surgical definition of a PSA cut-off value of 0.2 ng/mL is more sensitive than the Phoenix definition of PSA nadir plus 2 ng/mL is for detecting BCR [10, 11]. Different definitions of BCR make comparing the oncological outcomes between SEED-BT and RP difficult. To resolve this, we compared the BCR-free rate with the same definition of a PSA cut-off value of 0.2 ng/mL. Our results suggested that SEED-BT and RP had comparable BCR-free rates in this setting. Comparing the BCR-free rate between the two modalities using the surgical definition may be inadequate because PSA levels gradually decline after prostate SEED-BT and can take > 5 years to reach a nadir [18]. However, reaching a PSA value of < 0.2 ng/mL would be an important index for managing SEED-BT because patients who reach this PSA value are less likely to experience recurrence after SEED-BT [19, 20]. Morris et al. reported the reanalysis of androgen suppression combined with elective nodal and dose-escalated radiation therapy (the ASCENDE-RT trial) using the surgical definition of BCR in high- and intermediate-risk patients. Unlike dose-escalated EBRT, replacing the Phoenix definition with the surgical definition did not affect BCR after SEED-BT. These authors concluded that BCR after SEED-BT or RP can be directly compared using the surgical definition [21].

Differences in surgical techniques and the incidence of positive surgical margins also make comparing the oncological outcomes between SEED-BT and RP difficult [12]. Published data on positive surgical margins vary and depend on patient selection, the surgeons and the procedures [9, 22–24]. In the present study, approximately 53% of patients had an unfavorable intermediate risk, and 29.4% of RP patients had positive surgical margins. The incidence of positive surgical margins in patients who underwent open RP was higher than that of patients who underwent laparoscopic RP (34.7% vs. 22.5%). The higher rate of positive surgical margins adversely influenced BCR after RP and independently predicted BCR. Our incidence of positive surgical margins yielded comparable or slightly higher rates than those of previous studies [9, 23–25]. To eliminate the effects of surgical technique on clinical outcomes, we compared the effectiveness of the two treatments assuming that no RP patients had positive surgical margins. In this study, the BCR-free rates did not significantly differ. Advances in diagnostic imaging modalities and surgical techniques such as robot-assisted RP may decrease the positive-margin rates [12, 22, 25, 26]. However, obtaining no positive surgical margins in patients with intermediate-risk prostate cancer would be impossible because the incidence of pathological T3 exists to some extent by stage migration. The oncological outcomes after SEED-BT would be comparable even if the advanced procedure reduced the positive-margin rate to as close to zero as possible.

For RP, pathological findings from surgical specimens, including positive surgical margins, pathological T3–4, and Gleason grade groups, independently predict BCR or the use of salvage hormonal therapy. Predicting oncological outcomes from surgical specimens is considered as an advantage of RP over SEED-BT. However, for SEED-BT, we detected no risk factors for predicting BCR or the use of salvage hormonal therapy. The present study included SEED-BT alone and the combination of SEED-BT and EBRT. Although the 24.7% of patients who underwent combined therapy may have affected the analysis of these risk factors, the BCR-free rates did not significantly differ between SEED-BT alone and SEED-BT plus EBRT in our subgroup analysis. No consensus has been reached regarding the benefit of additional EBRT for patients with intermediate-risk prostate cancer [2, 27].

In addition to its retrospective nature, the present study had several limitations. First, our results lacked adequate power to generalize them with those of other institutions because this study represented the experiences of three tertiary centers. While overall survival rates did not significantly differ between the two treatments, our overall survival analysis was inadequate because we did not include factors related to life expectancy, such as co-morbidities and smoking history, in the propensity score matching. Survival data from multiple centers involving more patients with longer follow-up periods are needed. Second, no comparable applications for evaluating health-related quality of life were available in the present study. We did not evaluate patient-reported outcomes or erectile dysfunction between the two modalities. Third, pathological findings were not centrally reviewed. Additionally, we lacked information on prostate volume, nerve-sparing status, and individual surgeon volume. These variables were likely confounding factors influencing interpretation of the results. Finally, 30.3% of the SEED-BT patients received NHT for < 1 year. The short-term NHT may have affected the BCR rate.

## Conclusions

Treatment with either SEED-BT or RP had comparable oncological outcomes for patients with intermediate-risk prostate cancer for our median 8-year follow-up even when the BCR rate was calculated using the same definition of a PSA cut-off value of 0.2 ng/mL for both treatments. We believe that our results may help patients decide which treatment best suits their medical needs. Further evaluation is needed regarding the role of definitive treatments for patients with intermediate-risk prostate cancer undergoing robot-assisted RP or stereotactic radiotherapy.

## Supplementary Information

**Additional file 1.** **Table S1.** Causes of death in patients treated with SEED-BT and RP.

## Data Availability

The datasets generated and/or analyzed during the current study are not publicly available because the protocol did not include a data sharing plan; however, they are available from the corresponding author on reasonable request.

## Abbreviations

BCR: Biochemical recurrence
CI: Confidence interval
CTV: Clinical target volume
EBRT: External-beam radiotherapy
HR: Hazard ratio
NCCN: National Comprehensive Cancer Network
NHT: Neoadjuvant hormonal therapy
PSA: Prostate-specific antigen
PTV: Planning target volume
RP: Radical prostatectomy
SEED-BT: Seed brachytherapy
