# Supplementary material for: Direct comparison of low-dose-rate brachytherapy versus radical prostatectomy using the surgical definition of biochemical recurrence for patients with intermediate-risk prostate cancer
Source: Radiat Oncol. 2022 Apr 11;17:71. doi: 10.1186/s13014-022-02046-x (PMC8996640; doi:10.1186/s13014-022-02046-x)
Supplement: Supplementary file 1 — Additional file 1. Table S1. Causes of death in patients treated with SEED-BT and RP. [file 13014_2022_2046_MOESM1_ESM.docx]

Supplementary Table 1: Causes of death in patients treated with SEED-BT and RP

| Cause of death | SEED-BT (n=19) | RP (n=15) |
| --- | --- | --- |
| Malignant neoplasm |  |  |
| Cancer of unknown primary | 0 | 1 |
| Colon cancer | 1 | 0 |
| Esophageal cancer | 0 | 1 |
| Gastric cancer | 1 | 2 |
| Laryngeal cancer | 0 | 2 |
| Lung cancer | 1 | 0 |
| Pancreatic cancer | 2 | 1 |
| Prostate cancer | 1 | 0 |
| Other than malignant neoplasm |  |  |
| Accident | 1 | 1 |
| Cardiac disease | 1 | 0 |
| Cerebral hemorrhage | 2 | 0 |
| Subarachnoid hemorrhage | 0 | 1 |
| Interstitial pneumonitis | 1 | 1 |
| Pneumonitis | 1 | 2 |
| Septic shock | 1 | 0 |
| Unknown | 6 | 3 |

Abbreviations: SEED-BT, seed brachytherapy; RP, radical prostatectomy.
